# Pathway-based analysis of mutation impact

**DOI:** 10.1186/1753-6561-6-S6-O3

**Published:** 2012-10-01

**Authors:** Joshua Stuart

**Affiliations:** 1 University of California Santa Cruz, CA, USA

## 

Genomic damage plays an important role in cancer onset and progression. But how exactly do mutations, copy number changes, and epigenetic events affect the wiring of otherwise normal-functioning cellular pathways? Genomic probing with technologies such as RNA sequencing, copy number profiling, and DNA promoter methylation arrays are providing unprecedented views of cells. Uncovering predictive models from these datasets to shed light on key disrupted pathways in cancer is a major challenge that could offer breakthroughs for personalized medicine.

I'll describe a new pathway-based strategy that gives novel insight into the functional impact of mutations in particular genes. The major mechanism by which cancer arises is through somatic mutations. Individual tumors can contain hundreds to thousands of mutations. It is critical to distinguish mutations that have an important role defining the cancer - driver mutations - from mutations that are unimportant to the tumor - passenger mutations. A pathway-based approach is able to detect a shift in the downstream effects of an altered gene compared to what is expected from its upstream regulatory input. Application to several datasets across multiple tissues revealed several important driver mutations even among rarely mutated genes. Thus, pathway analysis shows promise in differentiating driver and passenger events that will increase our understanding of cancer disease mechanisms, which can help identify novel targets for treatment.

